# Antibiotic prescribing practices for acute respiratory illness in children less than 24 months of age in Kenema, Sierra Leone: is it time to move beyond algorithm driven decision making?

**DOI:** 10.1186/s12879-023-08606-0

**Published:** 2023-09-25

**Authors:** Troy D. Moon, Ibrahim Sumah, Gustavo Amorim, Foday Alhasan, Leigh M. Howard, Harriett Myers, Ann F. Green, Donald S. Grant, John S. Schieffelin, Robert J. Samuels

**Affiliations:** 1 Department of Tropical Medicine and Infectious Diseases, Tulane University School of Public Health and Tropical Medicine, 1440 Canal Street, Suite 2300, New Orleans, Louisiana 70112 USA; 2 Department of Pediatrics, Division of Pediatric Infectious Diseases, Tulane University School of Medicine, 1440 Canal Street, Suite 1600, New Orleans, Louisiana 70112 USA; 3 Vanderbilt Institute for Global Health, Vanderbilt University Medical Center, 2525 West End Avenue, Suite 750, Nashville, TN 37203 USA; 4 Kenema Government Hospital, Ministry of Health and Sanitation, 1 Combema Road, Kenema, Sierra Leone Sierra Leone; 5 Department of Biostatistics, Vanderbilt University Medical Center, 2525 West End Avenue, Suite 1000, Nashville, TN 37203 USA; 6 Department of Pediatrics, Division of Pediatric Infectious Diseases, Vanderbilt University Medical Center, D-7235 Medical Center North, 1161 21st Avenue South, Nashville, TN 37232 USA; 7 College of Medicine and Allied Health Sciences, University of Sierra Leone, New England Ville, Freetown, Sierra Leone

**Keywords:** Acute respiratory illness, Pediatrics, Antibiotics, Sierra Leone

## Abstract

**Background:**

Lower respiratory tract infections are the leading cause of mortality in young children globally. In many resource-limited settings clinicians rely on guidelines such as IMCI or ETAT + that promote empiric antibiotic utilization for management of acute respiratory illness (ARI). Numerous evaluations of both guidelines have shown an overall positive response however, several challenges have also been reported, including the potential for over-prescribing of unnecessary antibiotics. The aims of this study were to describe the antibiotic prescribing practices for children less than 24 months of age with symptoms of ARI, that were admitted to Kenema Government Hospital (KGH) in the Eastern Province of Sierra Leone, and to identify the number of children empirically prescribed antibiotics who were admitted to hospital with ARI, as well as their clinical signs, symptoms, and outcomes.

**Methods:**

We conducted a prospective study of children < 24 months of age admitted to the KGH pediatric ward with respiratory symptoms between October 1, 2020 and May 31, 2022. Study nurses collected data on demographic information, medical and medication history, and information on clinical course while hospitalized.

**Results:**

A total of 777 children were enrolled. Prior to arrival at the hospital, 224 children (28.8%) reported taking an antibiotic for this illness without improvement. Only 15 (1.9%) children received a chest radiograph to aid in diagnosis and 100% of patients were placed on antibiotics during their hospital stay.

**Conclusions:**

Despite the lives saved, reliance on clinical decision-support tools such as IMCI and ETAT + for pediatric ARI, is resulting in the likely over-prescribing of antibiotics. Greater uptake of implementation research is needed to develop strategies and tools designed to optimize antibiotic use for ARI in LMIC settings. Additionally, much greater priority needs to be given to ensuring clinicians have the basic tools for clinical diagnosis, as well as greater investments in essential laboratory and radiographic diagnostics that help LMIC clinicians move beyond the sole reliance on algorithm based clinical decision making.

## Background

Globally, lower respiratory tract infections (LRTI) are the leading cause of mortality in young children [1, 2]. Recent estimates by the World Health Organization (WHO) indicate that acute respiratory infection (ARI), principally pneumonia, causes approximately 1.4 million annual deaths in children under five-years of age [3]. The main bacterial causes of pneumonia in developing countries are S*treptococcus pneumoniae* and *Haemophilus influenzae* type B, [4, 5] while a growing proportion of LRTI has been attributed to viral causes such as *respiratory syncytial virus* (RSV), *human metapneumovirus* (hMPV), *parainfluenza virus* (PIV), *adenovirus (AdV)*, and *influenza virus* [6, 7]. It is estimated that children under five-years of age suffer between 3 and 6 episodes of symptomatic ARI each year, regardless of their country of residence or socio-economic situation [8, 9]. The prognosis of these infections may be worse in sub-Saharan Africa and other regions where children experience a high burden of co-morbid diseases including malnutrition, malaria, tuberculosis (TB), and human immunodeficiency virus (HIV) [10]. As national immunization programs for common bacterial causes of ARI have expanded in low- and middle-income countries (LMIC), the contribution of viral pathogens to ARI and LRTI has been increasingly recognized [11, 12]. However, clinicians continue to rely on guidelines that promote empiric antibiotic utilization for a broad range of conditions, some of which may be unnecessary to improve outcomes, and likely are contributing to increased antibiotic resistance as well as the development of severe immunologic, metabolic, and neurobehavioral adverse health outcomes in children [13].

Two of the most common guidelines for the management of children with respiratory symptoms in resource limited settings come from the Integrated Management of Childhood Illnesses (IMCI) and the Emergency Triage Assessment and Treatment Plus (ETAT+) [14, 15]. IMCI, developed by the WHO and UNICEF in the 1990’s, aimed to meet the objectives outlined in the Millennium Development Goals (MDGs) [16] and reduce morbidity and mortality of common and potentially serious childhood illnesses. The central focus of IMCI is identifying non-specific signs of serious illness and in managing conditions such as diarrhea/dehydration, malnutrition, fever, and cough/difficulty breathing [17, 18]. ETAT+, also developed by the WHO, is a complement manual to IMCI and was first published in 2005. ETAT + was designed to implement triage and management of common conditions in the setting of pediatric emergencies [19].

Numerous evaluations of both IMCI and ETAT + have reported on the overall positive response that these guidelines have had in the management of sick children and in their impact on helping to reduce under-five mortality globally. Positive impacts have included more accurate triaging of the child and reductions in the time taken for a sick child to be evaluated [20, 21]. Despite these positive impacts, several challenges have also been reported, including the potential for over prescribing of unnecessary antibiotics [22, 23]. Studies have shown that as many as 75% of children given antibiotics for presumed respiratory tract infections and over 90% of children given antibiotics for diarrheal illness, likely did not need them [22, 24, 25].

Sierra Leone, situated in West Africa, has a 2020 estimated population of roughly 7.9 million persons, of which more than a million live in the Western Area of Sierra Leone [26]. As of 2019, Sierra Leone was ranked 182 out of 189 countries on the United Nations Development Program (UNDP), Human Development Index (HDI) [27]. National economic development has been slowed by a civil war (1991–2002) and more recently by the 2014–2016 West African Ebola outbreak, which led to the loss of nearly 4,000 lives, of whom over 90 were health workers including 11 medical doctors [28].

Malaria, diarrhea, pneumonia, and other infections are the four main cause of mortality in children under-five in Sierra Leone [29, 30]. According to the Sierra Leone 2019 Demographic and Health Survey (DHS), there has been a gradual reduction in the under-five mortality rate. For example, mortality dropped from 140 deaths per 1,000 live births in 2008 to 122 deaths per 1,000 live births in 2019; and infant mortality (dying within the first year of life) decreased from 89 deaths per 1,000 live births in 2008 to 75 deaths per 1,000 live births in 2019. Nevertheless, Sierra Leone is still one of the countries with the highest under-five mortality rates. In order to address this, the “Free Health Care Initiative” was implemented in 2010, establishing free health services to pregnant women, lactating mothers, and children under-five throughout the country [20, 31].

Using IMCI or ETAT+, pneumonia is diagnosed based on the presence of cough and/or difficult or fast breathing. Current recommendations state that uncomplicated pneumonia should be treated with a minimum of 5-days of antibiotics and severe pneumonia should receive between 7 and 10 days [32]. However, conditions associated with wheezing (such as asthma), can also have associated symptoms of fast breathing and use of accessory muscles that could potentially lead the evaluating clinician towards a false decision of severe bacterial disease and subsequent antibiotic prescription, per the above guidelines.

The aims of this study were to describe the antibiotic prescribing practices for children less than 24 months of age with symptoms of ARI that were admitted to Kenema Government Hospital (KGH) in the Eastern Province of Sierra Leone, and to identify the number of children empirically prescribed antibiotics who were admitted to hospital with ARI as well as their clinical signs, symptoms, and outcomes.

## Methods

We conducted a prospective surveillance study of children admitted to the KGH pediatric ward between October 1, 2020 and May 31, 2022. Children were eligible for enrollment if they were < 24 months of age, had been admitted to the hospital less than 48-hours prior to enrollment, with or without fever of ≥ 38.0ºC, and presented with at least one of the following respiratory symptoms: cough (< 14 days duration); difficulty breathing (retractions, tachypnea for age: >50 breaths per minute for those 0–12 months of age and > 40 breaths per minute for those 13–23 months of age), and/or nasal flaring. Patients who did not meet the above criteria were excluded from participation.

KGH, located in Kenema District of Eastern Province, has a catchment area of roughly 600,000 persons and was at the epicenter of Sierra Leone’s efforts during the 2014–2016 West African Ebola outbreak [31, 33]. The pediatric ward admits ~ 400 children per month. The pediatric clinical staff includes one doctor, three community health officers, and ~ 40 nurses. KGH has X-ray machine capacity but no radiologist on site. Currently, requests for X-ray at KGH are based solely on clinician discretion and not based on any clinical guidelines. KGH lacks blood culture capacity or respiratory viral diagnostics. Laboratory capacity is limited to rapid tests for malaria, typhoid, syphilis, and HIV; microscopy for malaria and stool samples; and point-of-care testing for TB.

Children presenting to the emergency triage with respiratory symptoms were screened for potential eligibility by emergency care nurses. If determined eligible by the above inclusion criteria, parents or legal guardians were approached for enrollment. Study nurses collected data utilizing a paper-based study instrument which was subsequently uploaded into a password-protected, tablet-based, online database utilizing REDCap (Research Electronic Data Capture). This method allowed for the recording of demographic information, medical history including medications, and information on clinical course while hospitalized. Data quality control was conducted by study investigators who reviewed all completed paper-based study instruments and confirmed the accuracy of data entered into the electronic database.

Current guidelines instruct triage clinicians to first “*assess*” a child brought in with respiratory symptoms and then to go through several steps consisting of *ask, look, listen, check*, and *feel*.(20)^,^(21) The goal of this exercise is to identify children with rapid breathing or signs of respiratory distress such as nasal flaring or use of accessory muscles for breathing. Next, clinicians should “*classify*” that child in terms of disease severity, only prescribing antibiotics if they fall within the moderate or severe classifications [14, 15].

Admission diagnosis is made by the triage clinicians at the time of assessment and then reviewed/confirmed by a physician either at the time of admission, if present, or within the first 24 h of admission. Discharge diagnosis is made by the physician based on clinical course in the hospital and medical record review. Finally, study information collected related to discharge disposition focused on whether the patient was discharged alive, had died during hospitalization, or if they left the hospital against medical advice, and did not capture information related to cause of death.

Descriptive statistics were used to summarize the participants’ sociodemographic characteristics using frequencies and proportions (for dichotomous or categorical variables) or medians with interquartile ranges (IQR) for continuous variables.

## Results

From October 01, 2020 to May 31, 2022 a total of 1,778 children arrived at the emergency triage with respiratory symptoms and were screened for study eligibility. Of these, 1,037 were confirmed eligible per criteria and 253 (24%) were not enrolled due to parent/guardian refusal. Seven children were not included in this analysis due to incomplete data collected in the medical record. A total of 777 children < 24 months of age were enrolled during the study period and included in this analysis, of which 365 (47%) were female (Table 1). The median age of enrolled children was 9.2 months [IQR: 5.2, 14.3]. Maternal education for enrolled children was low, with 707 mothers (90%) having achieved only a secondary education or less. Household density was high with a median number of 8.0 persons per household [IQR: 5.0, 12.0]. Further, the median number of children less than five years of age living in each household was 2.0 [IQR: 1.0, 3.0]. About a third (30.6%) of enrolled children reported that another household family member had an ARI within the two weeks prior to admission to hospital. Additionally, 275 (35.4%) children came from households with at least one reported smoker, while very few [37 (4.8%)] reported coming from a household that utilized an indoor burning stove.

**Table 1:** Demographic characteristics of children hospitalized with respiratory symptoms, Kenema, Sierra Leone October 2020-May 2022

Variables (N = 777)	N (%)
Sociodemographic characteristics	
Sex	
Female	365 (47.0%)
Age in months (median [IQR])	9.2 [5.2, 14.3]
Mother’s education	
No education	253 (32.6%)
Primary education	92 (11.8%)
Secondary education	362 (46.6%)
Some or completed college	70 (9.0%)
Number of household members, (median [IQR])	8.0 [5.0, 12.0]
Number in household members aged < 5 years (median [IQR])	2.0 [1.0, 3.0]
Other household member with ARI* in 2 weeks prior to admission	238 (30.6%)
Smoker lives in the household	275 (35.4%)
Household uses an indoor burning stove	37 (4.8%)

*ARI = acute respiratory illness

Of our enrolled children, 769 (99%) reported having cough (< 14 days), while 700 (90.1%) reported fast breathing/shortness of breath. Signs of respiratory distress, such as nasal flaring or use of accessory muscles were reported in 201 (25.8%) children, of which roughly 50% were classified as being moderate to severe by the admitting clinician. Wheezing was documented in only 20 (2.6%) children, while other upper respiratory tract symptoms such as runny nose, congestion, and earache were reported in 53%, 44%, and 2.2% of children respectively. A malaria infection was confirmed in 365 (46.9%) children. Other conditions such as HIV, TB, and sickle cell were not common. Prior to arrival at the hospital, 224 children (28.8%) reported taking an antibiotic for this illness without improvement. Only 15 (1.9%) patients enrolled in our study received a chest radiograph to aid in diagnosis and 100% of patients were placed on antibiotics during their hospital stay (Table 2). Of the antibiotics reportedly taken prior to hospitalization, oral Amoxicillin was by far the most common (61.6%), followed by Trimethoprim/Sulfamethoxazole (14.7%), Metronidazole (10.3%), and injectable Ceftriaxone (8%) (Table 3**)**. More than 90% of patients (n = 700) were discharged alive, and the median length of hospital stay for all children was 4.0 days [IQR: 3.0, 6.0].

**Table 2:** Clinical characteristics for children less than 24 months admitted to hospital with respiratory symptoms

Variables (N = 777)	N (%)
Respiratory Tract Symptoms	
Either cough and/or Fast breathing / shortness of breath	777 (100.0%)
Cough	769 (99.0%)
Fast breathing / shortness of breath	700 (90.1%)
Nasal flaring / use of accessory muscles (n = 777)	201 (25.8%)
Moderate to severe nasal flaring / use of accessory muscles (n = 201)	104 (51.7%)
Wheezing	20 (2.6%)
Other respiratory tract symptoms	
Runny nose	412 (53.0%)
Nasal congestion	342 (44.0%)
Earache	17 (2.2%)
Gastrointestinal symptoms	
Poor Feeding	457 (58.8%)
Vomiting	427 (55.0%)
Diarrhea	245 (31.5%)
Other symptoms	
Fatigue / weakness	172 (22.1%)
Seizures	89 (11.5%)
Irritability / fussiness	14 (1.8%)
Skin Rash	75 (9.7%)
Other Diagnosis at admission	
Malaria	365 (46.9%)
HIV/AIDS	12 (1.5%)
Tuberculosis	7 (0.9%)
Sickle cell	11 (1.4%)
Antibiotics taken prior to hospitalization for this illness	224 (28.8%)
Antibiotics given during this hospitalization	777 (100.0%)
Chest radiograph performed this hospitalization	15 (1.9%)
Status at discharge (n = 771)	
Alive	700 (90.8%)
Dead	36 (4.7%)
Discharged against medical advice/abscond	35 (4.5%)
Length of stay in hospital in days (median [IQR])	4.0 [3.0, 6.0]

**Table 3:** Antibiotics given prior and during hospitalization

Variables	N (%)
Antibiotics given prior to hospitalization (n = 224)*	
Amoxicillin	138 (61.6%)
Trimethoprim/sulfamethoxazole	33 (14.7%)
Metronidazole	23 (10.3%)
Ceftriaxone	18 (8.0%)
Erythromycin	11 (5.0%)
Ampicillin	6 (2.7%)
Cefuroxime	5 (2.2%)
Azithromycin	1 (< 1.0%)
Unknown	5 (2.2%)
Antibiotics given during hospitalization (n = 777)*	
Ampicillin + Gentamicin	589 (75.8%)
Ceftriaxone	202 (26.0%)
Amoxicillin	115 (14.8%)
Ampicillin	75 (9.6%)
Ampicillin + Cloxacillin	64 (8.2%)
Erythromycin	46 (5.9%)
Metronidazole	41 (5.2%)
Amoxicillin/Clavulanate	15 (1.9%)
Azithromycin	9 (1.2%)
Cefotaxime	7 (< 1.0%)
Trimethoprim/sulfamethoxazole	5 (< 1.0%)
Clindamycin	3 (< 1.0%)
Cefuroxime	2 (< 1.0%)
Unknown	1 (< 1.0%)

*Percentages may add up to more than 100% as more than one medication could have been provided

**Fig. 1:**
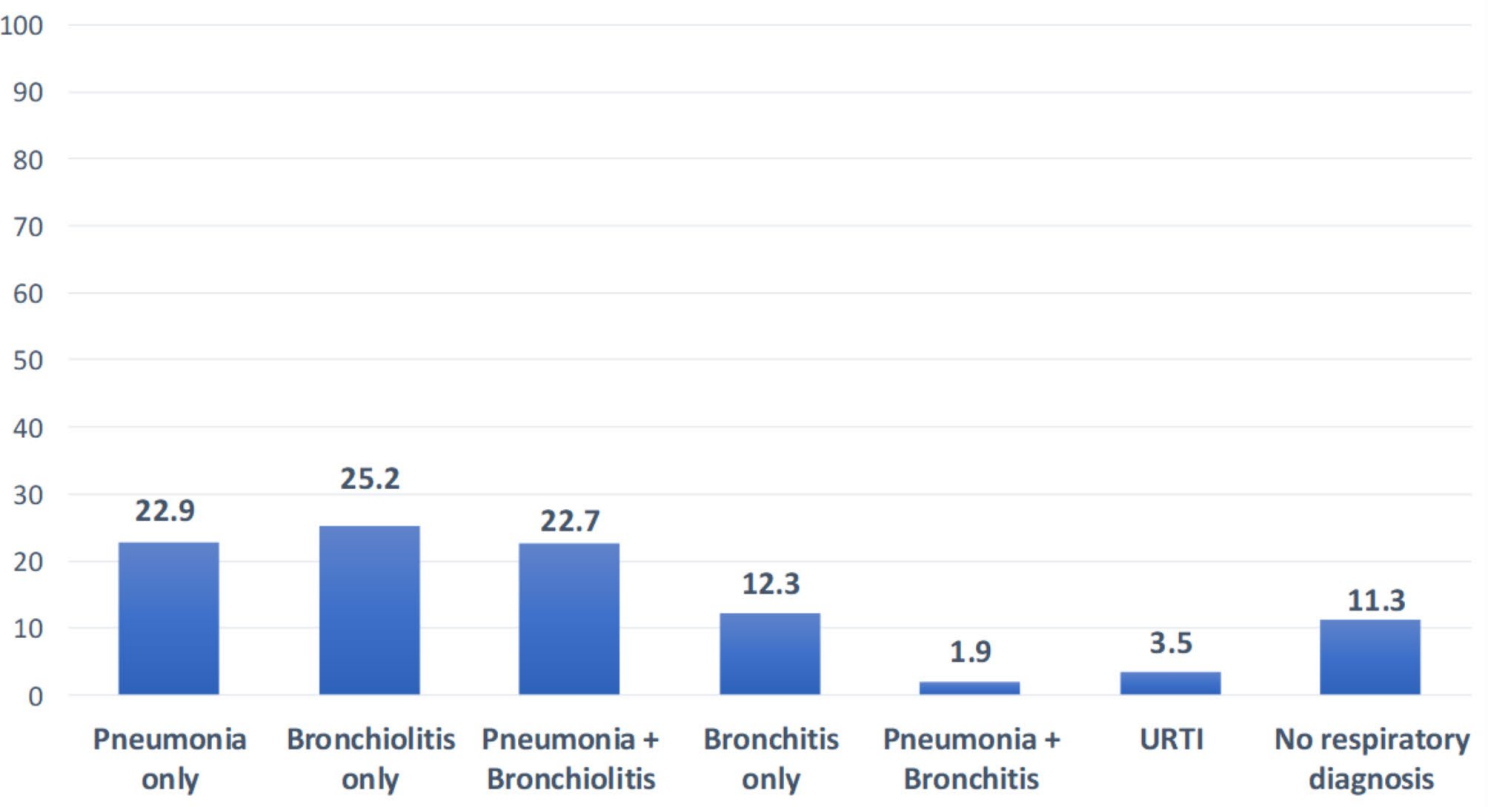
Percent of respiratory diagnoses at discharge given for children less than 24 months, admitted with respiratory symptoms *URTI = Upper Respiratory Tract Infection

At discharge, 689 (88.7%) enrolled children had at least one respiratory diagnosis listed as a contributing discharge diagnosis. The most common diagnoses given were bronchiolitis alone (25.2%); followed by pneumonia alone (22.9%); then pneumonia plus bronchiolitis (22.7%); and bronchitis only (12.3%) (Fig. 1). The antibiotics most commonly given during hospitalization were a combination of intravenous (IV) ampicillin + gentamicin (75.8%) and IV ceftriaxone (26%).

## Discussion

The goal of algorithms such as IMCI and ETAT + in the management of ARI is to identify those children with a higher likelihood of having a bacterial pneumonia based on their presentation with rapid breathing or signs of respiratory distress such as nasal flaring or use of accessory muscles for breathing.(14)(15) In our study population, 700 children (90%) were considered to have rapid breathing at initial triage. However, of these, only roughly 25% had documentation of nasal flaring and/or accessory muscle use; and of these, only approximately 50% were classified as being moderate to severe (n = 104, 13.3% of total population). The diagnosis of pneumonia can be complex and further confounded by the fact that pneumonia is often associated with viral detection [23].

Studies have shown that when utilized correctly, IMCI does a fairly good job of identifying children with life-threatening bacterial pneumonia, with a sensitivity reported as high as 96% [34, 35]. To date, the prevention of excess mortality from life-threatening bacterial pneumonia has been the desired outcome of use of decision-support tools such as IMCI. However, to the contrary, the specificity of IMCI has been reported as being relatively lower at 58%, [36] suggesting that upwards of 40%, or more, of children are over-diagnosed as having a severe bacterial infection and thus receiving treatments they likely do not need and that could be causing harm.

In post-study audits and debriefing conducted by our study team, it was generally felt that in situations like ours, where auxiliary laboratory and radiographic diagnostics are not yet existent or not fully utilized, triage clinicians do not have enough information (laboratory or radiographic results) at the time of initial assessment to confidently rule out severe bacterial infections and thus are defaulting to the provision of empiric antibiotics. Even when done correctly, appropriate antibiotic prescribing practices are not always sustained over time due to factors such as difficulties in accurately recognizing signs and symptoms of severe disease, parental pressure to prescribe antibiotics, or clinicians “just wanting to be on the safe side” [22, 23].

Per IMCI guidelines, during the initial assessment, wheezing should be assessed, and if present, a trial of up to three doses of rapid inhaled bronchodilators should be given. If the child responds positively, then a clinician could decide not to give antibiotics [14]. Wheezing is a symptom more commonly associated with a respiratory virus, especially RSV [37–39]. In our study population, only 20 children (2.6%) had documentation of wheezing in their medical record and only 15 children (1.9%) had documentation of a chest radiograph being performed, despite its availability at the hospital. Nonetheless, fully 100% of our study population received antibiotics, though we estimate that only approximately 45% had a discharge diagnosis that included pneumonia (see Fig. 1). The likely over-prescribing of antibiotics for pediatric ARI in our study is consistent with what has been seen elsewhere, though our results are still comparatively high [40, 41]. In our post-study audits, we found that the triage nurses, responsible for conducting the initial assessment of the child, did not readily have access to a stethoscope. Thus, it is possible that the small number of patients with documented wheezing in our study was not necessarily because wheezing was infrequent, but rather because the triage nurses did not have the basic tools necessary to fully realize the IMCI or ETAT + guideline assessment.

The strength of our study lies in the real-world context for which it was conducted, a rural resource-constrained setting that is representative of health facilities across Sierra Leone. However, our results should be interpreted within the context of the following limitations. First, our study population was limited to children under two years of age. As a result, our analysis does not represent the full range of children´s ages for which the IMCI and ETAT + guidelines were written, children less than five years of age. Next, some important clinical questions about the children enrolled in our study were unable to be analyzed due to limitations in the data we collected. For example, we were not able to determine if enrolled children were sicker and more likely to receive antibiotics than those children not enrolled. Further, while we did report on the participant´s hospital disposition such as discharged home alive, left against medical advice, or death, we were unable to elucidate what the causes of death were or describe the situation that led up to the child´s death. We highlight that our triage nurses lacked easy access to stethoscopes and as a result only a small number of children were documented as having wheezing in their medical record. Per the guidelines, children with wheezing that respond to bronchodilator therapy would not likely be prescribed antibiotics. However, without documentation of this very important pathway within the algorithm´s care cascade, we are left speculating as to whether the use of antibiotics in our study population were appropriate or not.

Decision-support tools such as IMCI and ETAT + are designed to help clinicians consistently deliver evidence-based care to their patients. The introduction of IMCI in the mid-1990s and ETAT + in 2005 have contributed greatly to country progress toward reductions in under-five mortality, as first strived for with the MDGs (1990–2015) and now with the Sustainable Development Goals (SDG, 2015–2030). Despite this, full realization of IMCI and ETAT+’s potential has been limited by a continued lack of basic resources such as stethoscopes for triage nurses, as seen in our study, or access to auxiliary laboratory and radiographic diagnostics that can help a clinician feel more confident in the care decisions they make, for example, when not to prescribe antibiotics. Our findings indicate that there is room for improvement in terms of adherence to these guidelines for management of pediatric ARI. Only a few children had documentation of wheezing in their chart and subsequent implementation of the guideline´s wheezing care cascade prior to starting antibiotics. Additionally, only roughly 13% of the children enrolled in our study were classified as having moderate or severe disease. In this context, simple resource investments ensuring access to basic clinician tools such as stethoscopes, as well as continuous educational training that reinforces auscultation and other physical exam skills, in addition to chest x-ray interpretation, are low-hanging fruit that have the potential to improve the identification and subsequent management of children with severe bacterial pneumonia.

## Conclusions

Despite the lives saved, reliance mainly on clinical decision-support tools such as IMCI and ETAT+, for pediatric ARI, is resulting in the likely over-prescribing of antibiotics, and as such, contributing towards the development of negative outcomes such as antibiotic resistance. While we do not advocate for the cessation in the use of these guidelines, we advocate for greater country-level and international investments in essential basic clinical, laboratory, and radiographic diagnostic tools that give clinicians in these low-resource settings more options from which to base their clinical decisions, thus reducing their contribution to the development of other problems, such as antimicrobial resistance, due to the practical real-world lack of precision that is occurring in the implementation of these guidelines. We propose that greater uptake of implementation research, in LMIC, is needed to develop strategies to fully realize IMCI and ETAT + implementation and to develop tools designed to optimize antibiotic use for ARI in these settings. Finally, a paradigm shift which demands, and invests in, standard of care practices that help LMIC clinicians move beyond the sole reliance on algorithm based clinical decision making is needed.

## Data Availability

All data generated or analyzed during this study are included in this published article.
